# Effect of gelatin treatment on tow deformation in resin-impregnated glass fiber

**DOI:** 10.1038/s41598-022-23569-z

**Published:** 2022-11-08

**Authors:** Mei-Xian Li, Yu Ren, Dasom Lee, MooSun Kim, SungWoong Choi

**Affiliations:** 1 School of Textile and Clothing, Nantong University, Nantong, China; 2 School of Mechanical and Aerospace Engineering, Seoul National University, Seoul, South Korea; 3 Urban Railroad Research Department, Korea Railroad Research Institute, Uiwang, South Korea; 4 Department of Mechanical System Engineering, Gyeongsang National University, Tongyeong, South Korea

**Keywords:** Engineering, Materials science

## Abstract

The potential use of gelatin materials in the liquid composite molding manufacturing (LCM) process was investigated, with specific focus on the reinforcement deformation phenomenon. The adoptability of gelatin as a binder in a composite material with glass fiber for application in the LCM process was evaluated by analyzing the permeability and microscopic structure of the gelatin-coated glass fiber. To assess the tow deformation, the permeability of the non-crimped unidirectional glass fiber mat was evaluated at different flow rates that could be applied in the LCM process. Hysteresis of the permeability was observed as the flow rate increased and decreased, indicative of tow deformation. The permeability of the gelatin-treated glass fiber mat exhibited a relatively smaller variation than that of the untreated glass fiber at the same flow rate. Tow deformation in the untreated and gelatin-treated non-crimped glass fiber mats at different flow rates was evaluated by microscopic analysis and quantified using the tow thickness index. Relatively smaller variations in the permeability and minimal changes in the tow thickness of the gelatin-treated glass fiber mat were observed via microscopic analysis, indicating that gelatin effectively maintained the binding structure of the glass fiber mat.

## Introduction

The continuing demand for the practical implementation of high-performance fibers in structures has led to a continuing interest in high-strength fiber-reinforced composites. Composite materials such as glass-fiber and carbon-fiber-reinforced composites have been widely used for structures and outfitting in the shipping, aircraft, and automotive industries^1^, where analyzing the relationship between the design characteristics and structural performance is necessary to ensure the safety of structural designs^2–4^. Reinforcement deformation problems have attracted the attention of researchers in the area of composite manufacturing. Reinforcement deformation problems in composite manufacturing are related to impregnation in the fiber, fiber pullout, debonding, and cavitation of the matrix during the composite molding process. However, a material that acts as a binder in the manufacturing process should prevent fiber deformation during the composite molding process.

The adoptability of gelatin in composite materials has been widely reported. Narbat et al.^5^ investigated the use of hydroxyapatite and gelatin composite scaffolds to mimic the mineral and organic composition of natural bones. Yan et al.^6^ produced poly (l-lactide acid)-blend‐gelatin (PLLA‐gelatin) nanofibers via electrospinning, whereas Wang et al.^7^ produced tubular scaffolds composed of polylactide fibers (outside layer) and silk fibroin-gelatin fibers (inner layer) via electrospinning. Balaji et al.^8^ fabricated three-dimensional scaffolds with a porous interconnected matrix using keratin, chitosan, and gelatin, where porous keratin–gelatin (KG) and keratin–chitosan (KC) composites were used as raw materials. The practical implementation of gelatin fibers as a cross-linked suturing material was explored by Nagura et al.^9^. Various approaches for producing gelatin fibers have been explored. Fan et al.^10^ produced alginate and gelatin blended fibers by spinning a solution of raw materials through a viscose‐type spinneret into a coagulating bath containing aqueous CaCl_2_ and ethanol. Kozlowska et al.^11^ developed three-dimensional collagen/gelatin/hydroxyethyl cellulose composites and microspheres loaded with gelatin and collagen/gelatin. The production and application of gelatin materials are still in their early stages. Further implementation relies on optimizing the material properties for specific applications.

Composites of carbon fibers and gelatin have been developed using solvent casting and solution‐impregnation techniques, where the mechanical properties (tensile strength and modulus, elongation at break, and shear strength) were adjusted based on the fiber volume fraction, glycerol (plasticizer) content, gelatin content, and fiber form^12^. Rodríguez-Castellanos et al.^13^ evaluated the use of hydrolyzed corn starch-gelatin as a base matrix with (5 wt%) and without cellulose fiber reinforcement to form containers via extrusion blow molding. Hanani et al.^14^ evaluated the mechanical and barrier properties of composite films manufactured by combining gelatin with corn oil, using a twin-screw co-rotating extruder. Furthermore, Zaman and Beg^15^ evaluated the fundamental properties of gelatin film-laminated polycaprolactone (PCL) bio-composites with varying gelatin contents and investigated the effect of gamma radiation after 2-ethylhexyl acrylate (EHA) pretreatment. The above studies demonstrate the potential applicability of gelatin in composite materials.

Moreover, gelatin material have been suggested as one of the effective binders in the composite material. Typically, Roh and Lee^16^ investigate the effect of gelatin coating for the multi-walled nanotube (MWNT) polypropylene (PP) composites with gelatin coated carbon fibers. They found that CNT particles can be minimized with gelatin acting as a binder in the carbon fiber. Guo et al.^17^ produced bioactive glass/gelatin composite scaffolds with different amounts of tetrapod zinc oxide whiskers showing morphology, mechanical properties and in vitro bioactivity of the composite scaffolds. Younes et al.^18^ developed Silica gel composites using four types of polymer binders. Among them, galatin was examined adoptability as polymer binder. Gautam et al.^19^ proposed to fabricate the composite nanofibrous tissue engineering-scaffold consisting of polycaprolactone and gelatin by electrospinning method showing the scaffold made from the combination of natural polymer (gelatin). Gareev et al.^20^ developed for the production of gelatin-based polymer composites with introduced particles of conductive polymers.

In addition, gelatin material may be a more effective material or binder in the manufacturing process or could improve the properties of composite materials which were attempted to adapt to the various field of composite material. Wan et al.^12,21^ reported a carbon-fiber-reinforced gelatin composite. The preparation and mechanical properties of the gelatin-based composites were investigated, and the mechanical properties were evaluated. Wan et al.^22^ also presented gelatin-based composites reinforced with short carbon fibers, woven fibers, and carbon fibers to examine the influence of the architecture of the reinforcement on the performance of gelatin-based composites. Khan^23^ investigated the morphological, mechanical, and thermal characteristics of jute-gelatin composites. Similarly, gelatin has been adopted in various fields of composite materials.

This study aims to explore the use of gelatin to identify appropriate materials minimizing reinforcement deformation in the composite manufacturing process. Specifically, the adoptability of gelatin in composite materials was examined in the liquid composite molding (LCM) manufacturing process, in order to address reinforcement deformation issues, with specific emphasis on tow deformation. The adoptability of gelatin in the composite material was experimentally investigated by analyzing the changes in the permeability of reinforcement. Tow deformation in the untreated and gelatin-treated non-crimped glass fiber mats at different flow rates was evaluated by microscopic analysis and quantified using the tow thickness index.

## Theory

LCM processes (typically, resin transfer molding and vacuum-infusion processes) are widely used to manufacture composites in a cost-effective manner. For fiber reinforcements impregnated with liquid resin, the resin flow in the LCM process can be explained using Darcy’s law:

1 $${u}_{i}=-\frac{{K}_{ij}}{\mu }\frac{\partial p}{\partial {x}_{j}}$$

where ${u}_{i}$*, K, μ,* and *p* represent the volume-averaged resin velocity, permeability tensor of the reinforcement, fluid viscosity, and fluid pressure, respectively.

In observing the flow behavior in the reinforcement (characterized by porous media), the permeability of the reinforcement was evaluated to understand the flow behavior. The permeability is usually determined experimentally by measuring the pressure difference between two distinct points at a given flow rate (*Q*, mm^3^/s) under steady-state flow conditions where the reinforcement is fully saturated in the resin flow.

2 $${K}_{ij}=\frac{\mu \frac{Q}{A}}{\left(-\frac{dP}{dx}\right)}=\mu \frac{Q}{A}\frac{L}{\left({P}_{1}-{P}_{2}\right)}$$

Here, ${K}_{ij}$, *L*, *P*_*1*_, and *e* are the permeability (m^2^), distance between the two distinct points, and fluid pressure at points 1 and 2, respectively.

Generally, a constant permeability value is adopted in the composite molding, regardless of the flow and reinforcement deformation. However, different values for the saturated and unsaturated permeability have been reported^24–29^. This may be attributed to reinforcement distortion and deformation during the LCM process. The same value of saturated, unsaturated permeability is related to the assumption that fiber tows was not deformed during the resin impregnation^30^.

## Experimental materials and method

### Materials

Gelatin may be an effective material for incorporation into composite materials to enhance composite molding processes. Gelatin has many excellent characteristics as a binder such as excellent biocompatibility^31^, good adhesion and dispersion abilities^32^ compare to other kind of binder material.

**Figure 1:**
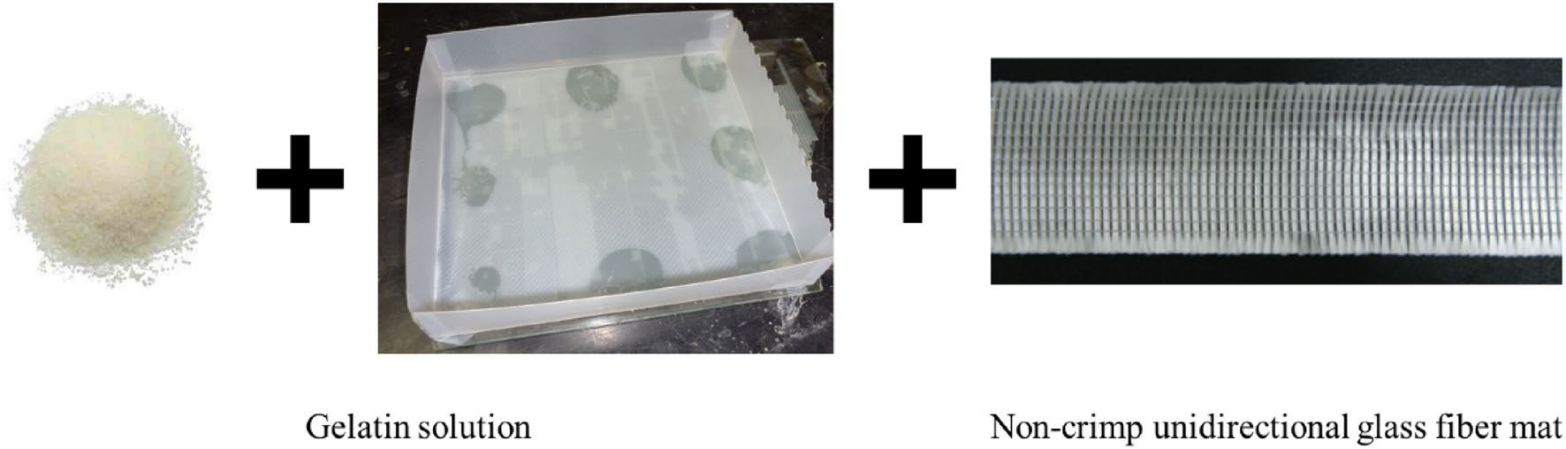
Gelatin treatment of non-crimped unidirectional glass fiber mat.

To examine the adaptability of gelatin to the composite material, gelatin was coated onto a non-crimped (NC) unidirectional glass fiber mat. Gelatin was obtained by thermal denaturation of collagen. The gelatin formed during collagen degradation in the sol state in a warm aqueous solution^33^ is shown in Fig. 1. At temperatures below 20 °C, gelatin changed from the sol state to the gel state; glass fiber mats were coated with aqueous solutions of gelatin, thereby facilitating the use of gelatin as a liquid-type binder. The characteristics of the gelatin used in this study (250 Bloom, Geltech Co., Ltd., Korea) are listed in Table 1. For reinforcement, a non-crimped (NC) unidirectional glass fiber mat (Owens Corning, USA) was used, as shown in Fig. 1. The non-crimped glass fiber mat consisted of E-glass fabric, where each tow consisted of 1000 filaments with diameters of 16.5 μm. The horizontal and vertical dimensions of the tow cross-section were 1.5 and 0.5 mm, respectively. A polyester knit yarn (JBY denier) was used to maintain the structure of the fibers. Silicone oil (KF-96, Shin-Etsu, Japan) was used as the working fluid; the properties of fluid are listed in Table 2.

**Table 1:** Properties of gelatin.

Gelatin				
Product type	Gel strength (bloom)	Viscosity at 25 °C (mPa s)	Transparency (10% aqueous solution)	Application
250 Bloom	240–260	30–40	> 88%	Food products e.g. jelly and marshmallow

**Table 2:** Properties of working fluid, silicone oil.

Silicone oil		
Specific gravity at 25 °C	Viscosity at 25 °C (Pa s)	Surface tension (mN/m)
0.965	0.0765	20.9

### Experimental method

**Figure 2:**
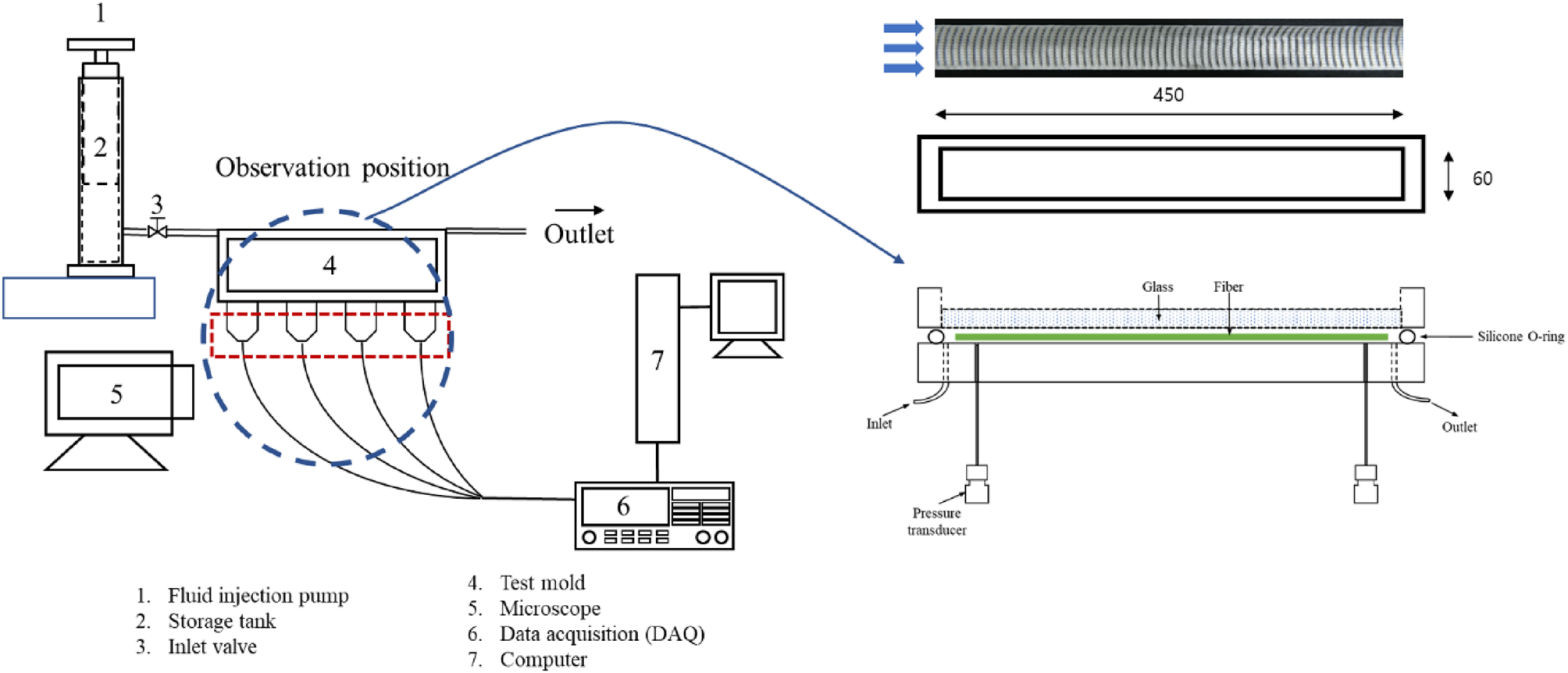
Schematic of experimental apparatus for observing tow behavior in double-scale porous media (all dimensions are in mm).

The NC glass fiber mat was immersed in 100 ml of 15 wt% aqueous gelatin solution (sol state) at 60 °C for 5 h. The glass fiber mat was dried in a drying oven at 100 °C until all the water evaporated to confirm the absence of residual water, allowing the gelatin to coat the surface of all glass fibers in the entire tow. The gelatin-treated glass fiber mat was cut to the size required to be placed in the mold. A schematic of the experimental apparatus is shown in Fig. 2. The NC glass fibers were prepared at a volume fraction of 50 wt% in the rectangular mold. The upper mold comprised 25 mm thick tempered glass, allowing observation of the fiber tow behavior during fluid flow from above. At the bottom part of the mold, the pressure of the fluid at each point was measured using pressure transducers (Sensys, Korea) along the flow direction at 120 mm intervals from the inlet. The pressure transducer has a measurement range of 0–0.1 MPa and a sensor accuracy of 0.030%; the pressure was measured using a data logger (Keithley 2700, US). To observe the tow deformation under constant flow conditions, silicone oil was injected at various constant flow rates (50, 100, 200, and 400 mm^3^/s) using a fluid injection pump. The fiber tow behavior was observed in real time using an optical microscope (Olympus Optical Co., Ltd., Japan).

## Results and discussion

### Permeability of NC glass fiber mat reinforcement

The permeability was evaluated to observe the hydrodynamic effects on the NC glass fiber mat reinforcement under constant flow rate conditions. In previous studies^30^, permeability measurements were conducted using a NC glass fiber mat under various viscous fluid conditions. In the present study, the permeability of the NC glass fiber mat was measured at different flow rates that could be applied to the LCM process.

**Figure 3:**
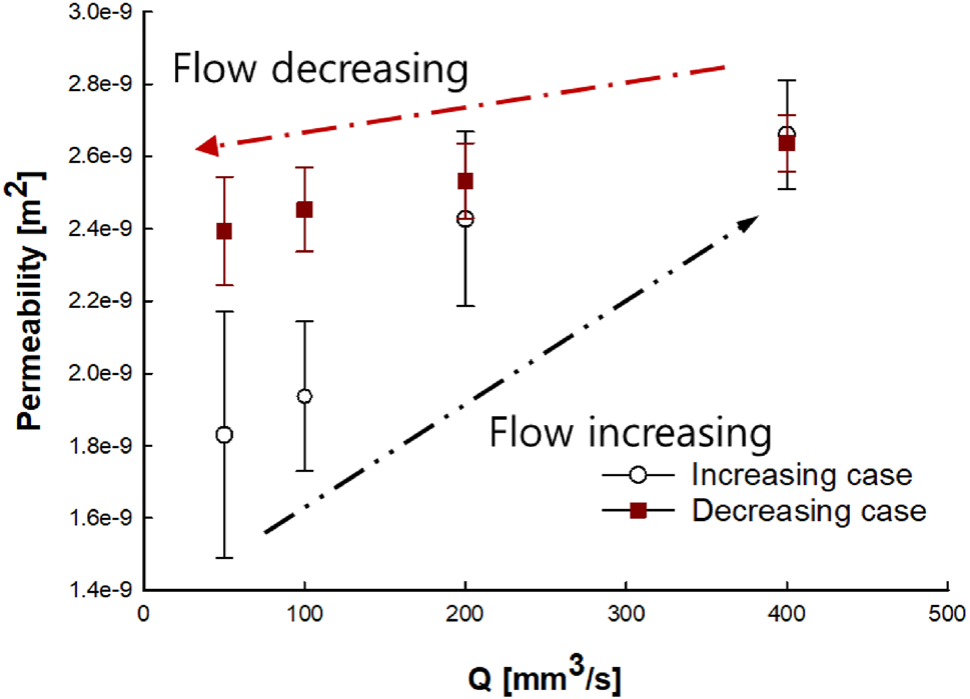
Change in permeability of untreated NC glass fiber mat at flow rates of 50, 100, 200, and 400 mm^3^/s.

At various constant flow rates (50, 100, 200, and 400 mm^3^/s), the permeability (m^2^) of the NC glass fiber mat was calculated using Eq. (2) by applying the pressures measured under each condition. The results are shown with error bars that indicate differences between the data points. For the untreated NC glass fiber mat (Fig. 3), the permeability increased or decreased depending on the flow rate. The permeability increased by 45% as the flow rate increased from 50 to 400 mm^3^/s and decreased by 10% as the flow rate decreased from 400 to 50 mm^3^/s; the decrease was less pronounced than the increase. It can be assumed that the constant values of the saturated and unsaturated permeability are related to the undeformed fiber tows during resin impregnation in the mold-filling process. However, plausible reasons for the observed permeability variation under different flow rate conditions have been reported, such as the void effect and fiber tow deformation problems^34,35^. Tow deformation during the mold filling process is the focus of the current study as one of the contributors to this discrepancy.

**Figure 4:**
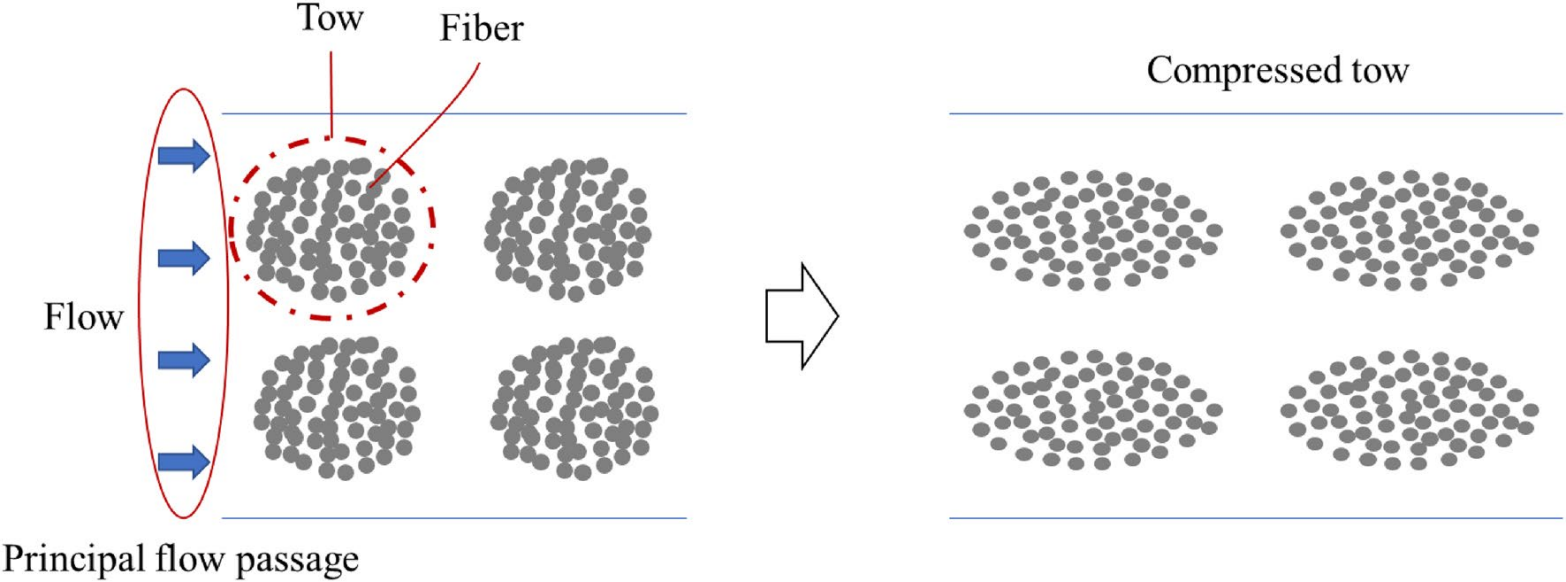
Schematic of tow compression and flow passage change.

During fluid flow in the NC glass fiber mat, the hydrodynamic force exerted on the fiber tows led to compression in the flow direction, thereby causing fiber tow deformation. The two compressions caused changes in the flow passage, where the increased flow rate widened the principal flow passage between the fiber tows, as shown in Fig. 4. Therefore, a higher flow rate led to increased permeability. As the flow rate decreased, a reversible process, in which the compressed tows return to their original shape, is generally expected to be observed as the hydrodynamic fluid force decreases^30^. However, the deformed tows did not return to their original shape when the hydrodynamic force was reduced with decreasing flow rate, as shown in Fig. 3. Therefore, the permeability followed different trends under increasing and decreasing flow rate conditions, showing an irreversible characteristic known as permeability hysteresis. This hysteresis is attributed to tow deformation, which occurred as the flow rate changed, where the structural network in the fiber tow changed owing to altered flow passages from the hydrodynamic force of the fluid^30,36^.

**Figure 5:**
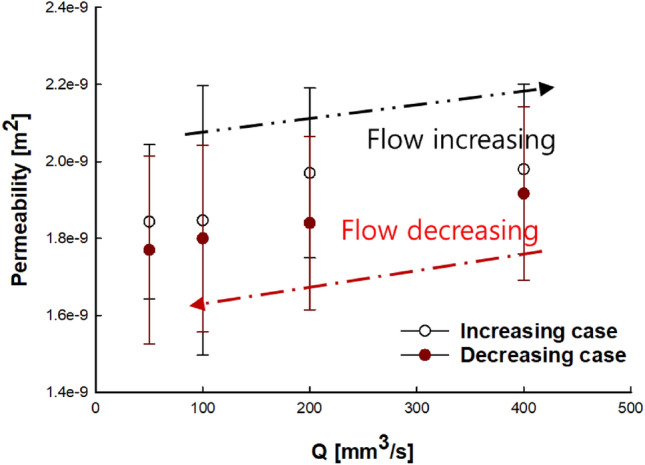
Permeability values at flow rates of 50, 100, 200, and 400 mm^3^/s for the gelatin-treated NC glass fiber mat.

The effect of gelatin treatment on the NC glass fiber mat was evaluated by permeability measurements; the results are shown in Fig. 5.

The permeability of the gelatin-treated NC glass fiber increased by 7% as the flow rate increased from 50 to 400 mm^3^/s, and decreased by 6% as the flow rate decreased from 400 to 50 mm^3^/s, where the permeability decrease was also less pronounced than the increase. The gelatin-treated NC glass fiber mat exhibited a relatively smaller variation in the permeability compared to the untreated NC glass fiber mat under the same conditions of increasing and decreasing flow rates: for the untreated NC glass fiber mat, there was a 45% increase in the permeability at flow rates between 50 and 400 mm^3^/s and a 10% decrease as the flow rate decreased.

Yoo et al.^37^ measured the permeability of woven carbon fabrics to compare the binder effect. When a non-reactive epoxy binder was used for the carbon fabric, the fiber permeability was significantly reduced in the neat fabric (about 15.5, 28.0% for the K_1_ and K_2_ direction) compared to the binder-treated fabric (about 12.4, 6.3% for the K_1_ and K_2_ direction). This result was consistent with the above explanation.

The measured permeability values reflect the effects of gelatin treatment on the NC glass fiber mat, where gelatin exerted a binding effect on the fiber tows. The effect of gelatin treatment was also confirmed by microscopic observation of the tow deformation.

### Microscopic observation

Microscopic examination of the appearance of the deformed tow can provide important information on the deformation of the tow at different flow rates. The effect of gelatin treatment on the NC glass fiber mat was observed using an optical microscope during fluid flow in the experimental mold, as shown in Fig. 2. The representative index, the tow thickness value, was measured for twenty samples under each condition using cross-sections of the deformed tow at different positions of the mold from the inlet.

**Figure 6:**
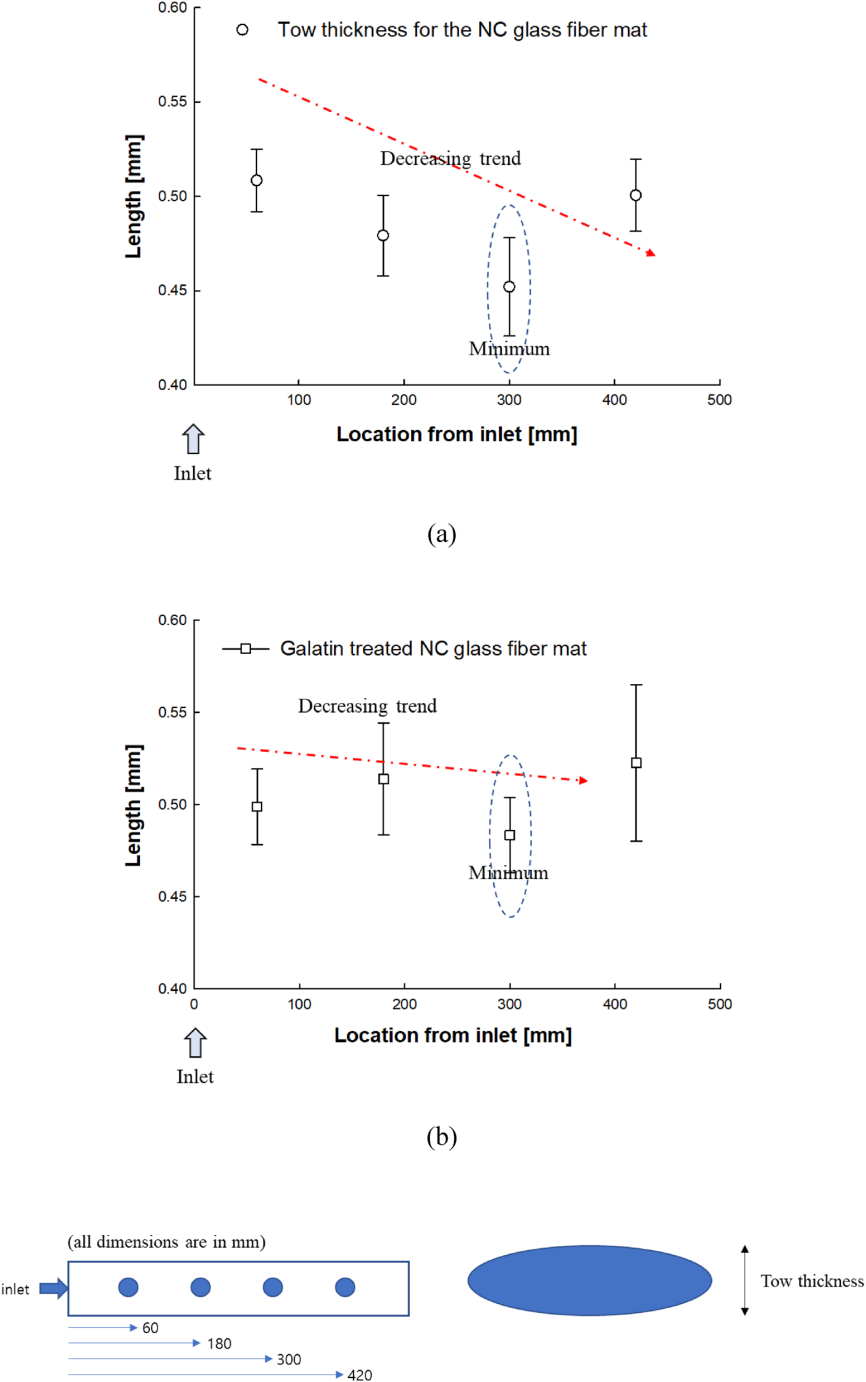
Tow thickness at cross-sections of the deformed tow at different positions of mold: untreated NC glass fiber mat (**a**), gelatin-treated NC glass fiber mat (**b**).

The tow thickness of the untreated and gelatin-treated NC glass fiber mats under constant flow rates (200 mm^3^/s) is shown with error bars in Fig. 6. For the untreated NC glass fiber mat, it is important to note that the tow thickness decreased along the flow position. In addition, the greatest decrease in the tow thickness was observed at a distance of 300 mm from the inlet. During resin impregnation in the mold filling process, the hydrodynamic fluid force affected the tow with fiber filaments. In addition, the developing area affecting the hydrodynamic force was approximately 300 mm from the inlet. Although the stitching threads maintained the tows in position, the shape of the tows changed between the stitching threads, thereby shape variation across the width of the fabric was observed. Therefore, the tow thickness decreased along the flow position.

**Figure 7:**
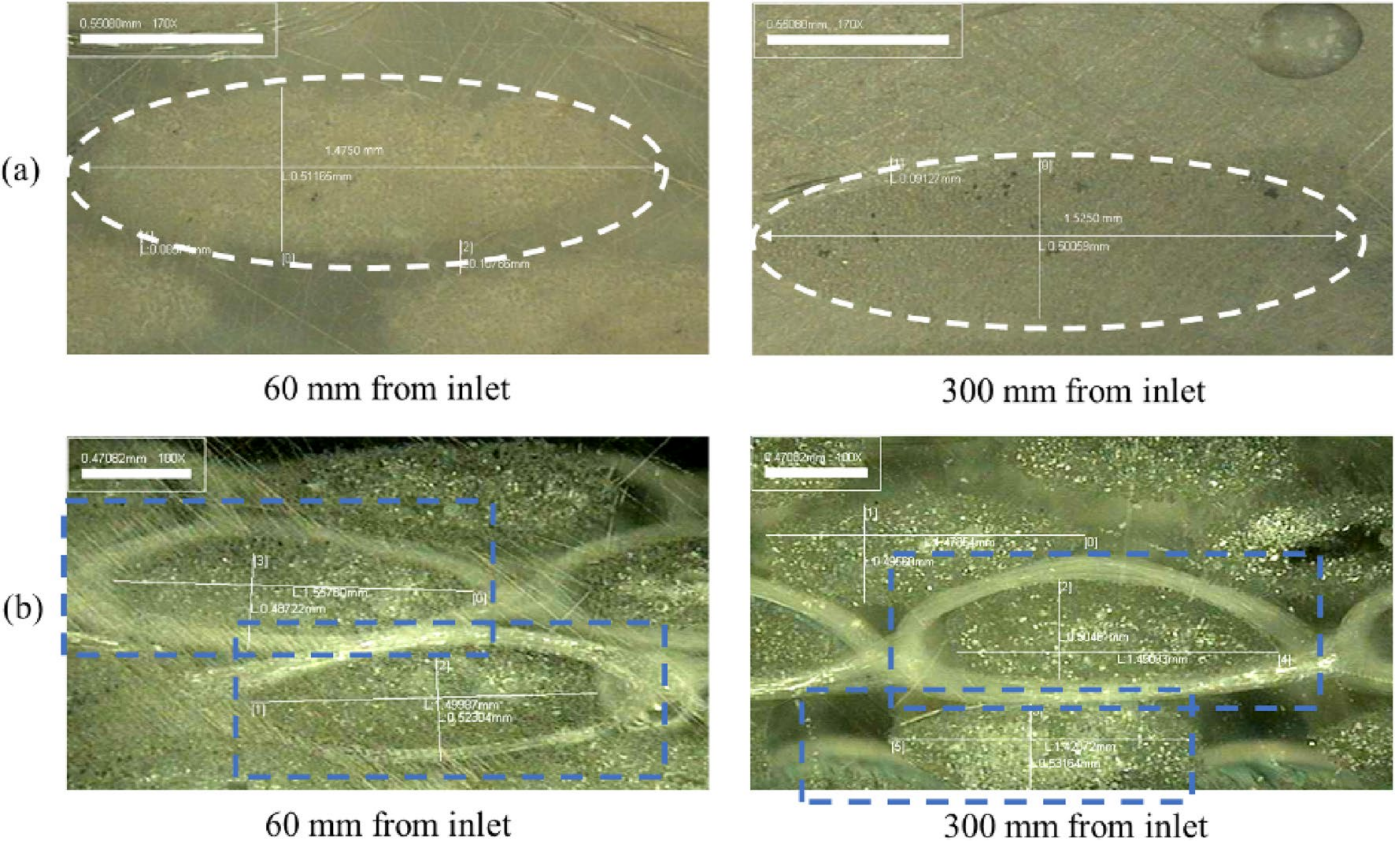
Tow behavior in (**a**) untreated and (**b**) gelatin-treated NC glass fiber mat at different positions.

**Figure 8:**
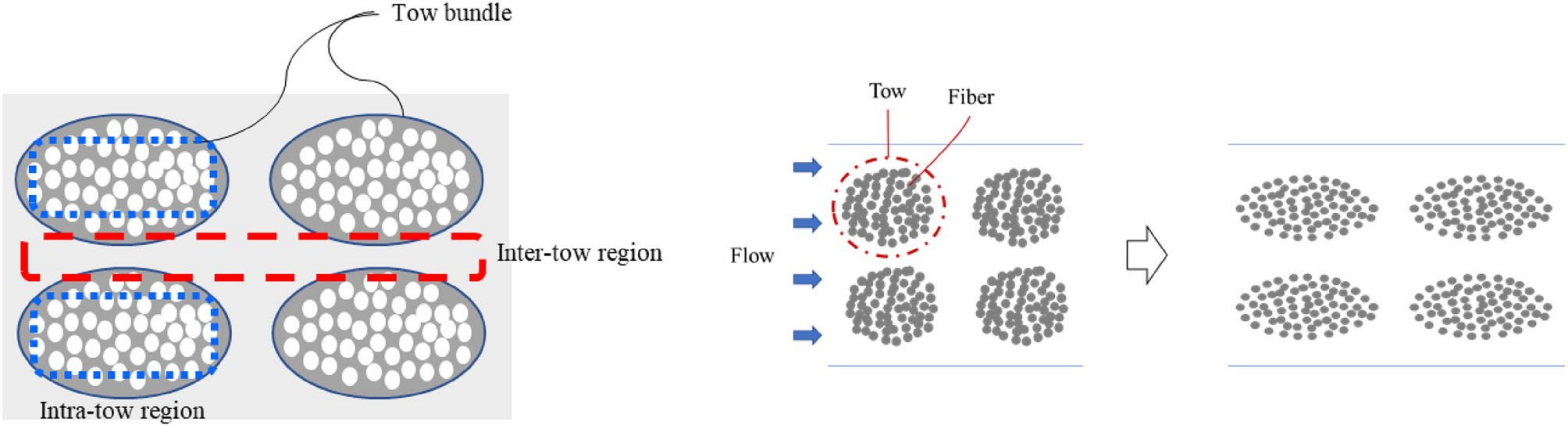
Inter-tow region and intra-tow region (left), tow behavior with flow in the double-scale porous media (right).

However, for the gelatin-treated NC glass fiber, a relatively small variation in the tow thickness was observed. Figure 7 shows representative microscopic images of the tow in the fiber bundle with transverse sections of fiber tows at different positions in the mold, where deformed tows were apparent in the fiber bundles. Tow fibers and fibers between the tows were observed, indicating intra-tow and inter-tow regions, respectively, as shown in Fig. 8^30,34^. For the main flow region in the double-scale porous media through the reinforcement, most of the fluid passes through the inter-tow regions, and the remainder passes through the intra-tow region^30,34^. The flow dominated in the inter-tow region, and the tow bundles became more elliptical as the flow rate increased^30,34^, as illustrated in Fig. 7. From the microscopic images of the tow, gelatin was bound to the fiber tow. Therefore, relatively smaller variations in the permeability and little variation in the tow thickness were observed for the gelatin-treated NC glass fiber mat compared to the untreated NC glass fiber mat.

Overall, gelatin treatment of NC glass fibers was demonstrated to be effective for binding the fiber tows based on the permeability measurement and microscopic examination of the deformed tow. Knit yarn in an NC glass fiber mat can serve as a binder for fiber tows and is commonly used to maintain the reinforcement structure within the material. However, knit damage may occur in the fiber, whereas gelatin treatment can provide an effective alternative binding effect to preserve the fiber structure.

## Conclusion

The adoptability of gelatin in composite materials via the liquid composite molding (LCM) manufacturing process was experimentally examined by monitoring the permeability and structure of the composite through microscopic analysis. The main conclusions are as follows:

1. The permeability of the NC glass fiber mat was measured at different flow rates that could be applied in the LCM process, where the permeability changed with increasing and decreasing trends as the flow rate changed.
2. During fluid flow in the NC glass fiber mat, the hydrodynamic force exerted on the fiber tows led to compression in the flow direction, thereby causing fiber tow deformation. Compression in the flow direction caused changes in the flow passage, and different flow rates led to differences in the permeability.
3. Differences in the permeability of the fiber mat were observed with increasing and decreasing flow rates, where the permeability changed in an irreversible manner, known as permeability hysteresis.
4. The permeability of the gelatin-treated NC glass fiber mat exhibited a relatively smaller variation than that of the untreated NC glass fiber mat under the same conditions of increasing and decreasing flow rate. Gelatin exerted a binding effect on the fiber tows.
5. Microscopic analysis of the untreated NC glass fiber mat showed that the tow thickness decreased along the flow position. Furthermore, the greatest decrease in the tow shape was observed at a distance of 300 mm from the inlet. However, for the gelatin-treated NC glass fiber, a relatively small variation in the tow thickness was observed.
6. Gelatin treatment of NC glass fibers was demonstrated to be an effective way to bind the fiber tows, based on permeability measurement and microscopic examination of the tow deformation.
7. The present findings regarding gelatin treatment of NC glass fibers can be effectively applied in a wide range of LCM processes for the development of composite materials. Future research will focus on investigating the effect of gelatin treatment on the material properties of NC glass fiber.

## Data Availability

The data that support the findings of this study are available from the corresponding author upon reasonable request.
